# Low birth weight leads to obesity, diabetes and increased leptin levels in adults: the CoLaus study

**DOI:** 10.1186/s12933-016-0389-2

**Published:** 2016-05-03

**Authors:** François R. Jornayvaz, Peter Vollenweider, Murielle Bochud, Vincent Mooser, Gérard Waeber, Pedro Marques-Vidal

**Affiliations:** Service of Endocrinology, Diabetes, and Metabolism, Lausanne University Hospital, Lausanne, Switzerland; Department of Medicine, Department of Internal Medicine, Lausanne University Hospital, Lausanne, Switzerland; Institute of Social and Preventive Medicine (IUMSP), Lausanne, Switzerland; Department of Medical Biology, Lausanne University Hospital, Lausanne, Switzerland

**Keywords:** Birth weight, Diabetes, Body composition, Leptin, Adiponectin, Cross-sectional study

## Abstract

**Background:**

Low birth weight is associated with increased rates of obesity, insulin resistance and type 2 diabetes, but the precise mechanisms for this association remain unclear. We aimed to assess the relationships between birth weight and markers of glucose homeostasis or obesity in adults.

**Methods:**

Cross-sectional population-based study on 1458 women and 1088 men aged 35–75 years living in Lausanne, Switzerland. Birth weight was self-reported and categorized into ≤2.5, 2.6–3.5, 3.6–4.0 and >4.0 kg. Body composition was assessed by bioimpedance. Leptin and adiponectin levels were measured by ELISA.

**Results:**

Women with low birth weight (≤2.5 kg) had higher levels of fasting plasma glucose, insulin, HOMA, diabetes and metabolic syndrome; a non significant similar trend was seen in men. In both genders, height increased with birth weight, whereas a U-shaped association was found between birth weight and body mass index, waist circumference and body fat percentage. After adjusting for age, smoking status, physical activity and fat mass, an inverse association was found between leptin and birth weight categories: adjusted mean ± standard error 17.3 ± 0.7, 16.2 ± 0.3, 15.6 ± 0.5 and 14.0 ± 0.8 ng/dL for birth weight categories ≤2.5, 2.6–3.5, 3.6–4.0 and >4.0 kg, respectively, in women (p < 0.05) and 9.8 ± 0.8, 9.1 ± 03, 7.8 ± 0.4 and 7.7 ± 0.5 ng/dL in men (p < 0.05). An inverse association was also found between reported birth weight and leptin to fat mass ratio: mean ± standard error 0.77 ± 0.04, 0.73 ± 0.02, 0.69 ± 0.03 and 0.62 ± 0.04 in women (p < 0.05); 0.46 ± 0.05, 0.45 ± 0.02, 0.39 ± 0.02 and 0.38 ± 0.03 in men (p < 0.05). No differences in adiponectin levels were found between birth weight groups.

**Conclusions:**

Middle-aged adults born with a low weight present a higher prevalence of diabetes and obesity and also higher leptin levels and leptin to fat mass ratio than adults born with a normal weight. The higher leptin levels and leptin to fat mass ratio among adults born with a low weight might be related to nutritional factors during childhood or to the development of leptin resistance and/or higher leptin production by body fat unit. Subjects born with a low weight should be counselled regarding the risks of developing diabetes and/or cardiovascular disease.

**Electronic supplementary material:**

The online version of this article (doi:10.1186/s12933-016-0389-2) contains supplementary material, which is available to authorized users.

## Background

Low birth weight is associated with increased rates of obesity, insulin resistance and type 2 diabetes [1], which are components of the metabolic syndrome [2]. Albeit the precise mechanisms for this association remain unclear, metabolic changes that could be related to alterations in body composition can be found already in childhood [3]. For instance, people with low birth weight present with higher plasma leptin concentrations than would be expected from their degree of obesity [4, 5]. In children born with a low birth weight, leptin levels are increased after catch-up growth during childhood [6], but it is unclear what happens during later life. Further, it has been suggested that blood cord adiponectin levels are positively related with birth weight and BMI in newborns [7], with a subsequent increase in the risk of type 2 diabetes [8]; still, this statement has also been challenged [9]. Overall, whether leptin and adiponectin mediate the effects of low birth weight on obesity, insulin resistance and type 2 diabetes is unclear.

Altogether, to our knowledge, no study has ever assessed the associations between birth weight, diabetes and fat mass, and leptin or adiponectin levels in middle age people. Hence, in this study, we assessed the associations between birth weight, markers of glucose homeostasis, obesity, leptin and adiponectin levels using a cross-sectional, population-based study conducted in Lausanne, Switzerland.

## Subjects and methods

### Study population

The CoLaus Study is a prospective population-based study aimed at assessing the prevalence and deciphering the molecular determinants of cardiovascular risk factors in the population of Lausanne, Switzerland, a town of 117,161 inhabitants, of which 79,420 are of a Swiss nationality. Its sampling procedure has been described previously [10]. Briefly, the complete list of Lausanne inhabitants aged 35–75 years was provided by the population registry of the city. A simple, non-stratified random sample of 35 % of the overall population was drawn. Caucasian origin was defined as having both parents and grandparents born in a restricted list of countries (available from the authors). The following inclusion criteria were applied: (a) written informed consent; (b) aged 35–75 years and (c) willingness to take part in the examination and donate blood sample. Recruitment for the baseline survey began in June 2003 and ended in May 2006.

### Clinical data

All participants attended the outpatient clinic of the University Hospital of Lausanne in the morning after an overnight fast. Data were collected by trained field interviewers in a single visit lasting about 60 min.

Body weight and height were measured with participants standing without shoes in light indoor clothes. Body weight was measured in kilograms to the nearest 100 g using a Seca^®^ scale, which was calibrated regularly. Height was measured to the nearest 5 mm using a Seca^®^ height gauge. BMI was defined as weight (kg) divided by height (m) squared. Overweight and obesity were defined as previously described [11]. Waist circumference was measured with a non-stretchable tape over the unclothed abdomen at the mid-point between the lowest rib and the iliac crest. Two measures were made and the mean (expressed in cm) used for analyses. Abdominal obesity was defined as a waist circumference ≥88 cm for women and ≥102 cm in men.

Fat and fat-free mass (in percent of total body weight) were assessed by electrical bioimpedance in the lying position after a 5-min rest using the Bodystat^®^ 1500 body mass analyzer (Bodystat Ltd, Isle of Man, England). This device has been shown to correlate well (r = 0.968) with measurements from dual energy X-ray absorptiometry (DEXA) [12]. In a subset of 794 women who were also screened for osteoporosis using DEXA, the correlation between fat mass estimated by bioimpedance and DEXA was 0.852 (p < 0.001), with only a slight overestimation by BIA relative to DEXA (+0.9 kg). Participants had to be fasting, avoid strenuous physical activity during the previous 12 h and abstain from consuming caffeine or alcohol-containing beverages during 24 h before the analysis. All metallic adornments were removed, and measurement was performed after a 10-min rest in the lying position. The electrodes were positioned in the right side of the body according to the manufacturer’s instructions. Care was taken to ensure that the participants did not touch any metallic component of the bed and that the inner part of the thighs did not touch each other. Results were expressed as (%BF) and body fat mass was calculated as weight x %BF and expressed in kg.

Weight at birth was asked to all participants and expressed in kg. Four birth weight categories were created: ≤2.5, 2.6–3.5, 3.6–4.0 and >4.0 kg. No information regarding gestational age at birth, term or preterm birth was collected. Smoking status was classified as never, former and current smokers. A participant was considered as physically active if he/she practiced at least twice per week leisure-time physical activities with a minimal duration of 20 min. Menopausal status in women was defined by a positive answer to the question “are you already menopaused?”.

### Biological data

Venous blood samples (50 ml) were drawn in the fasting state. Fasting plasma glucose and insulin were measured, and the homeostasis model assessment index of insulin resistance (HOMA) was calculated [13]. High HOMA index was defined if >2.6. Diabetes was defined as fasting plasma glucose ≥7.0 mmol/L or presence of oral or injectable hypoglycaemic treatment. Metabolic syndrome was defined using the Third Adult Treatment Panel (ATP-III) criteria [2]. Total adiponectin was assessed by ELISA (R&D Systems, Inc., Minneapolis, USA), with a maximum inter-assay coefficient of variation (CV) of 8.3 % and a maximum intra-assay CV of 8.3 %. Leptin was also assessed by ELISA (American Laboratory Products Company, Windham, USA) with a maximum inter-assay CV of 12.8 % and a maximum intra-assay CV of 5.8 %.

### Statistical analyses

Statistical analyses were conducted using Stata v.14 (Stata Corp, College Station, TX, USA) and stratified by gender. Descriptive results were expressed as number of participants and (percentage) or as mean ± standard deviation. Bivariate analysis was conducted using Chi square test for categorical variables and using Student’s t test or one-way analysis of variance (ANOVA) for quantitative values. For continuous variables, post hoc pairwise comparisons using the method of Scheffe were performed when the results of the ANOVA were statistically significant. Multivariable analysis was conducted using logistic regression for binary dependent variables, and the results were expressed as odds-ratio and (95 % confidence interval); for quantitative variables, multivariable ANOVA was used and the results were expressed as adjusted mean ± standard error. The leptin to fat mass ratio was computed as previously described [14, 15]. Because leptin and adiponectin concentrations are skewed, natural log transformation was performed prior to analysis.

### Ethics, consent and permissions

The CoLaus Study was approved by the Institutional Ethics Committee of the University of Lausanne. All participants gave their signed informed consent before entering the study.

## Results

### Selection of participants

Of the initial 6733 participants, 2546 (37.8 %) reported information on birth weight and were thus included in the study. The characteristics of the included and excluded participants are summarized in Additional file 1: Table S1.

### Sample’s characteristics

Clinical characteristics according to gender are summarized in Table 1. Age was similar between women and men. Women reported lower tobacco consumption, had a lower BMI and higher body fat levels and were less frequently overweight and obese than men. Women also reported lower birth weights and had higher leptin and adiponectin levels than men. Of the 2546 participants included in the analysis, 2404 (94.4 %) were considered as Caucasian.

**Table 1:** Clinical characteristics of participants, by gender

	Women (n = 1458)	Men (n = 1088)	p value
Age (years)	50.2 ± 10.1	49.7 ± 9.9	0.197
BMI (kg/m^2^)	24.5 ± 4.8	26.4 ± 3.9	<0.001
BMI categories (%)			<0.001
Normal	907 (62.2)	429 (39.4)	
Overweight	375 (25.7)	491 (45.1)	
Obese	176 (12.1)	168 (15.4)	
Waist circumference (cm)	82.0 ± 12.4	95.0 ± 11.2	<0.001
Abdominal obesity (%)	409 (28.1)	269 (24.8)	0.062
Body fat (%)	33.0 ± 8.0	22.9 ± 6.0	<0.001
Physically active (%)	855 (58.6)	611 (56.2)	0.210
Menopausal status (%)	632 (43.4)	NR	
Smoking status			<0.001
Never	694 (47.6)	385 (35.4)	
Former	384 (26.3)	384 (35.3)	
Current	380 (26.1)	319 (29.3)	
Birth weight (kg)	3.3 ± 1.0	3.5 ± 0.7	<0.001
Birth weight categories (%)			<0.001
≤2.5 kg	174 (11.9)	73 (6.7)	
2.6–3.5 kg	891 (61.1)	556 (51.1)	
3.6–4.0 kg	264 (18.1)	266 (24.5)	
>4.0 kg	129 (8.9)	193 (17.7)	

Results are expressed as mean ± standard deviation or number of people and (percentage). Normal weight was defined as a body mass index (BMI) < 25 kg/m^2^; abdominal obesity was defined as a waist circumference ≥88 cm for women and ≥102 cm in men. Between-gender comparisons performed using Student’s t test or Chi square

*NR* not relevant

### Birth weight and obesity markers

Bivariate associations between anthropometric markers and birth weight group are summarized in Table 2 (for women) and Table 3 (for men). All anthropometric markers showed significant differences between birth weight categories in both genders, with the exception of total fat mass in men (Table 3). Conversely, leptin levels did not differ between birth weight categories.

**Table 2:** Distribution of anthropometric and biological variables according to birth weight, women, unadjusted

	Birth weight categories (kg)				p value
	−2.5] (n = 174)	]2.5–3.5] (n = 891)	]3.5–4.0] (n = 264)	]4.0+ (n = 129)	
Anthropometry					
Height (cm)	161 ± 1^a^	164 ± 1^b^	165 ± 1^c^	166 ± 1^c^	<0.001
Weight (kg)	64.7 ± 1.0^a^	64.6 ± 0.4^a^	69.2 ± 0.8^b^	69.9 ± 1.1^b^	<0.001
BMI (kg/m^2^)	25.0 ± 0.4^a, b^	24.1 ± 0.2^a^	25.4 ± 0.3^b^	25.5 ± 0.4^b^	<0.001
Waist circumference (cm)	83.7 ± 0.9^a, b^	80.8 ± 0.4^a^	83.4 ± 0.8^b^	84.6 ± 1.1^b^	<0.001
Fat (% of body weight)	34.4 ± 0.6^a^	32.3 ± 0.3^b^	33.5 ± 0.5^a, b^	34.1 ± 0.7^a, b^	0.002
Fat mass (kg)	22.9 ± 0.7^a, b^	21.4 ± 0.3^a^	24.1 ± 0.6^b^	24.4 ± 0.8^b^	<0.001
BMI categories (%)					<0.001
Normal	98 (56.3)	592 (66.4)	150 (56.8)	67 (51.9)	
Overweight	48 (27.6)	220 (24.7)	68 (25.8)	39 (30.2)	
Obesity	28 (16.1)	79 (8.9)	46 (17.4)	23 (17.8)	
Abdominal obesity (%)	57 (32.8)	220 (24.7)	83 (31.4)	49 (38.0)	0.002
Adipokines					
Leptin (ng/dL)	18.1 ± 0.9	15.4 ± 0.4	16.9 ± 0.7	15.9 ± 1.1	0.230^§^
Leptin/fat mass ratio	0.77 ± 0.04^a^	0.72 ± 0.02^a, b^	0.71 ± 0.03^a, b^	0.61 ± 0.04^b^	0.035
Adiponectin (μg/dL)	11.8 ± 0.7	12.2 ± 0.3	11.9 ± 0.5	12.5 ± 0.8	0.612^§^
Markers of glucose homeostasis					
Fasting glucose (mmol/L)	5.46 ± 0.06^a^	5.24 ± 0.03^b^	5.28 ± 0.05^a, b^	5.30 ± 0.07^a, b^	0.013
Fasting insulin (μU/mL)	8.5 ± 0.4	7.3 ± 0.2	8.4 ± 0.3	6.9 ± 0.5	0.003
HOMA	2.10 ± 0.12	1.76 ± 0.05	2.05 ± 0.10	1.68 ± 0.14	0.004
Diabetes (%)	11 (6.3)	17 (1.9)	7 (2.7)	6 (4.7)	0.007
High HOMA (%)	35 (24.3)	117 (16.5)	37 (18.5)	15 (15.2)	0.133
Metabolic syndrome (%)	39 (22.4)	113 (12.7)	37 (14.0)	21 (16.3)	0.009

Results are expressed as number of people and (column percentage), as average ± standard deviation. Normal weight was defined as a body mass index <25 kg/m^2^; abdominal obesity was defined as a waist circumference ≥88 cm for women and ≥102 cm in men. Between-group comparisons performed using Chi square for categorical variables and by ANOVA for continuous variables. For continuous variables, post hoc pairwise comparisons using the method of Scheffe were performed when the results of the ANOVA were statistically significant; results with a different subscript are significantly different at p < 0.05 (corrected for multiple comparisons). For fasting insulin and HOMA, no pairwise difference at p < 0.05 (corrected for multiple comparisons) was found

^§^Statistical analysis performed on log-transformed data

**Table 3:** Distribution of anthropometric and biological variables according to birth weight, men, unadjusted

	Birth weight categories (kg)				p value
	−2.5] (n = 73)	]2.5–3.5] (n = 556)	]3.5–4.0] (n = 266)	]4.0+ (n = 193)	
Anthropometry					
Height (cm)	175 ± 1^a, c^	175 ± 1^a^	178 ± 1^b, c^	179 ± 1^b^	<0.001
Weight (kg)	80.8 ± 1.5^a, c^	80.0 ± 0.6^a^	84.5 ± 0.8^b, c^	87.0 ± 0.9^b^	<0.001
BMI (kg/m^2^)	26.3 ± 0.5^a^	26.0 ± 0.2^a^	26.8 ± 0.2^b^	27.2 ± 0.3^b^	<0.001
Waist circumference (cm)	94.8 ± 1.3^a, b^	93.8 ± 0.5^b^	95.9 ± 0.7^a, b^	97.4 ± 0.8^a^	<0.001
Fat (% of body weight)	23.5 ± 0.7	22.5 ± 0.3	23.2 ± 0.4	23.6 ± 0.4	0.089
Fat mass (kg)	19.3 ± 0.9^a, b^	18.4 ± 0.3^b^	20.1 ± 0.5^a, b^	21.0 ± 0.5^a^	<0.001
BMI categories (%)					0.004
Normal	28 (38.4)	248 (44.6)	99 (37.2)	54 (28.0)	
Overweight	33 (45.2)	236 (42.5)	120 (45.1)	102 (52.9)	
Obesity	12 (16.4)	72 (13.0)	47 (17.7)	37 (19.2)	
Abdominal obesity (%)	15 (20.6)	124 (22.3)	68 (25.6)	62 (32.1)	0.043
Adipokines					
Leptin (ng/dL)	10.0 ± 1.0	8.6 ± 0.4	8.2 ± 0.5	8.7 ± 0.6	0.635^§^
Leptin/fat mass ratio	0.46 ± 0.05	0.45 ± 0.02	0.39 ± 0.02	0.38 ± 0.03	0.097
Adiponectin (μg/dL)	8.1 ± 0.7	7.2 ± 0.2	7.1 ± 0.4	7.3 ± 0.4	0.848^§^
Markers of glucose homeostasis					
Fasting glucose (mmol/L)	5.80 ± 0.14	5.64 ± 0.05	5.67 ± 0.07	5.60 ± 0.08	0.655
Fasting insulin (μU/mL)	10.0 ± 0.9	9.5 ± 0.3	9.0 ± 0.5	9.5 ± 0.5	0.745
HOMA	2.79 ± 0.3	2.45 ± 0.11	2.42 ± 0.15	2.50 ± 0.18	0.712
Diabetes (%)	9 (12.3)	33 (5.9)	17 (6.4)	11 (5.7)	0.202
High HOMA (%)	18 (29.5)	139 (29.8)	63 (27.9)	46 (27.7)	0.938
Metabolic syndrome (%)	17 (23.3)	134 (24.1)	64 (24.1)	61 (31.6)	0.183

Results are expressed as number of people and (column percentage) or as average ± standard deviation. Normal weight was defined as a body mass index <25 kg/m^2^; abdominal obesity was defined as a waist circumference ≥88 cm for women and ≥102 cm in men. Statistical analysis by Chi square for categorical variables and by ANOVA for continuous variables. For continuous variables, post hoc pairwise comparisons using the method of Scheffe were performed when the results of the ANOVA were statistically significant; results with a different subscript are significantly different at p < 0.05 (corrected for multiple comparisons)

^§^Statistical analysis performed on log-transformed data

The results of the multivariate analysis of the associations between anthropometric markers and birth weight categories are summarized in Table 4 (for women) and Table 5 (for men). The inverse associations between birth weight and height or weight persisted in both genders. In women, both low and high birth weights were associated with a lower likelihood of presenting with normal weight in adulthood, while birth weights >3.5 kg were associated with a higher likelihood of developing abdominal obesity (Table 4). In men, a similar but nonsignificant inverse association was found between low birth weight and likelihood of presenting with normal weight in adulthood, and similar associations between birth weights >3.5 kg and normal weight or abdominal obesity were found (Table 5). A significant inverse association was also found between reported birth weight and the leptin to fat mass ratio in both women and men, while no differences in adiponectin levels were found between birth weight groups (Tables 4, 5). Sensitivity analysis restricting to people reporting a birth weight > 1.5 kg led to similar findings (Additional file 1: Tables S2a, S2b).

**Table 4:** Distribution of anthropometric and biological variables according to birth weight, women, multivariate adjusted

	Birth weight categories (kg)				p value	Linear	Quadratic
	−2.5] (n = 174)	]2.5–3.5] (n = 891)	]3.5–4.0] (n = 264)	]4.0+ (n = 129)			
Anthropometry^a^							
Height (cm)	161 ± 1	164 ± 1	165 ± 1	166 ± 1	<0.001	<0.001	0.069
Weight (kg)	64.5 ± 1	64.7 ± 0.4	69.2 ± 0.8	69.8 ± 1.1	<0.001	<0.001	0.797
BMI (kg/m^2^)	24.8 ± 0.4	24.1 ± 0.2	25.4 ± 0.3	25.4 ± 0.4	<0.001	0.086	0.285
Waist circumference (cm)	83.1 ± 0.9	81.0.0 ± 0.4	83.5 ± 0.7	83.9 ± 1.1	0.002	0.259	0.125
Fat (% of body weight)	33.8 ± 0.6	32.5 ± 0.2	33.6 ± 0.5	33.2 ± 0.6	0.061	0.861	0.421
Fat mass (kg)	22.4 ± 0.7	21.6 ± 0.3	24.1 ± 0.6	23.8 ± 0.8	<0.001	0.033	0.692
Normal weight	*0.70 (0.50–0.98)*	1 (ref.)	*0.66 (0.50–0.88)*	*0.59 (0.40–0.86)*		0.197	0.089
Abdominal obesity	1.35 (0.94–1.94)	1 (ref.)	*1.40 (1.03–1.90)*	*1.67 (1.12–2.49)*	–	0.206	0.106
Adipokines							
Leptin (ng/dL)^a^	17.8 ± 0.9	15.5 ± 0.4	16.9 ± 0.7	16.0 ± 1.1	0.357^§^	0.337^§^	0.522^§^
Leptin (ng/dL)^b^	16.9 ± 0.6	16.3 ± 0.3	15.8 ± 0.5	13.8 ± 0.8	0.008^§^	0.004^§^	0.241^§^
Leptin (ng/dL)^c^	16.7 ± 0.7	16.1 ± 0.3	16.1 ± 0.6	14.4 ± 0.8	0.147^§^	0.050^§^	0.341^§^
Leptin (ng/dL)^d^	17.3 ± 0.7	16.2 ± 0.3	15.6 ± 0.5	14.0 ± 0.8	0.012^§^	0.003^§^	0.541^§^
Leptin/fat mass ratio^b^	0.77 ± 0.04	0.73 ± 0.02	0.69 ± 0.03	0.62 ± 0.04	0.029	0.004	0.655
Adiponectin (µg/dL)^a^	11.6 ± 0.7	12.3 ± 0.3	11.9 ± 0.5	12.2 ± 0.8	0.560^§^	0.244	0.867
Adiponectin (µg/dL)^b^	11.8 ± 0.7	12.1 ± 0.3	12.2 ± 0.5	12.5 ± 0.8	0.433^§^	0.105^§^	0.796^§^
Adiponectin (µg/dL)^c^	11.9 ± 0.7	12.1 ± 0.3	12.2 ± 0.5	12.5 ± 0.8	0.594^§^	0.150^§^	0.800^§^
Adiponectin (µg/dL)^d^	11.7 ± 0.6	12.1 ± 0.3	12.2 ± 0.5	12.5 ± 0.8	0.406^§^	0.090^§^	0.970^§^
Markers of glucose homeostasis							
Fasting Glucose (mmol/L)^b, e^	5.37 ± 0.05	5.28 ± 0.02	5.26 ± 0.04	5.18 ± 0.06	0.115	0.016	0.903
Fasting Insulin (μU/mL)^b, e^	8.2 ± 0.4	7.5 ± 0.2	8.0 ± 0.3	6.5 ± 0.4	0.012	0.008	0.194
HOMA^b, e^	1.99 ± 0.1	1.83 ± 0.04	1.95 ± 0.08	1.50 ± 0.12	0.006	0.004	0.106
Diabetes^b^	*2.43 (1.02–5.79)*	1 (ref.)	0.75 (0.27–2.12)	1.40 (0.50–3.98)	–	0.285	0.053
Diabetes^c^	2.38 (0.97–5.84)	1 (ref.)	0.89 (0.32–2.48)	1.86 (0.65–5.29)	–	0.634	0.042
Diabetes^d^	*2.70 (1.13–6.45)*	1 (ref.)	0.71 (0.25–2.03)	1.17 (0.38–3.55)	–	0.134	0.067
High HOMA^b^	1.32 (0.83–2.12)	1 (ref.)	0.81 (0.51–1.28)	0.63 (0.33–1.19)	–	0.031	0.947
Metabolic syndrome^a^	*1.75 (1.15–2.68)*	1 (ref.)	1.12 (0.74–1.69)	1.05 (0.62–1.79)	–	0.140	0.195

Results are expressed as adjusted mean ± standard error or as odds ratio and (95 % confidence interval). Normal weight was defined as a body mass index <25 kg/m^2^; abdominal obesity was defined as a waist circumference ≥88 cm for women and ≥102 cm in men. Statistical analysis conducted using analysis of variance for continuous variables and logistic regression for categorical variables. Statistically significant odds ratios are indicated in italics. Column p value corresponds to the p value of the overall association test; column linear trend corresponds to the p value for testing a linear trend. Adjusted for: ^a^ age, smoking status and physical activity; ^b^ age, smoking status, physical activity and BMI; ^c^ age, smoking status, physical activity and waist circumference; ^d^ age, smoking status, physical activity and fat mass. Also adjusted for ^e^ antidiabetic drug treatment

^§^Statistical analysis performed on log-transformed data

**Table 5:** Distribution of anthropometric and biological variables according to birth weight, men, multivariate adjusted

	Birth weight categories (kg)				p value	Linear	Quadratic
	−2.5] (n = 73)	]2.5–3.5] (n = 556)	]3.5–4.0] (n = 266)	]4.0+ (n = 193)			
Anthropometry^a^							
Height (cm)	175 ± 1	176 ± 1	178 ± 1	179 ± 1	<0.001	<0.001	0.306
Weight (kg)	80.6 ± 1.5	80.0 ± 0.5	84.6 ± 0.8	86.9 ± 0.9	<0.001	<0.001	0.147
BMI (kg/m^2^)	26.2 ± 0.4	26.0 ± 0.2	26.8 ± 0.2	27.1 ± 0.3	<0.001	0.024	0.328
Waist circumference (cm)	94.8 ± 1.2	93.7 ± 0.4	96.1 ± 0.6	97.3 ± 0.8	<0.001	0.022	0.168
Fat (% of body weight)	23.7 ± 0.6	22.4 ± 0.2	23.3 ± 0.3	23.4 ± 0.4	0.018	0.946	0.075
Fat mass (kg)	19.4 ± 0.8	18.3 ± 0.3	20.2 ± 0.4	20.9 ± 0.5	<0.001	0.035	0.117
Normal weight	0.75 (0.45–1.26)	1 (ref.)	*0.71 (0.52–0.96)*	*0.47 (0.33–0.68)*	–	0.055	0.043
Abdominal obesity	0.90 (0.48–1.70)	1 (ref.)	1.27 (0.89–1.82)	*1.72 (1.17–2.53)*	–	0.039	0.610
Adipokines							
Leptin (ng/dL)^a^	10.0 ± 1.0	8.6 ± 0.3	8.2 ± 0.5	8.7 ± 0.6	0.649^§^	0.544^§^	0.209^§^
Leptin (ng/dL)^b^	10.1 ± 0.8	9.0 ± 0.3	8.0 ± 0.4	7.9 ± 0.5	0.088^§^	0.039^§^	0.534^§^
Leptin (ng/dL)^c^	10.1 ± 0.8	9.0 ± 0.3	8.0 ± 0.4	7.8 ± 0.5	0.055^§^	0.031^§^	0.721^§^
Leptin (ng/dL)^d^	9.8 ± 0.8	9.1 ± 0.3	7.8 ± 0.4	7.7 ± 0.5	0.016^§^	0.020^§^	0.625^§^
Leptin/fat mass ratio^b^	0.46 ± 0.05	0.45 ± 0.02	0.39 ± 0.02	0.38 ± 0.03	0.041	0.078	0.942
Adiponectin (µg/dL)^a^	8.1 ± 0.7	7.2 ± 0.2	7.1 ± 0.4	7.3 ± 0.4	0.875^§^	0.875^§^	0.745^§^
Adiponectin (µg/dL)^b^	8.1 ± 0.7	7.1 ± 0.2	7.1 ± 0.4	7.4 ± 0.4	0.906^§^	0.827^§^	0.639^§^
Adiponectin (µg/dL)^c^	8.1 ± 0.7	7.1 ± 0.2	7.1 ± 0.4	7.5 ± 0.4	0.880^§^	0.820^§^	0.592^§^
Adiponectin (µg/dL)^d^	8.1 ± 0.7	7.1 ± 0.2	7.2 ± 0.4	7.4 ± 0.4	0.898^§^	0.838^§^	0.577^§^
Markers of glucose homeostasis							
Fasting Glucose (mmol/L)^b, e^	5.64 ± 0.11	5.65 ± 0.04	5.68 ± 0.06	5.62 ± 0.07	0.927	0.930	0.673
Fasting Insulin (μU/mL)^b, e^	9.5 ± 0.8	9.8 ± 0.3	8.8 ± 0.4	9.1 ± 0.5	0.199	0.434	0.894
HOMA^b, e^	2.50 ± 0.25	2.55 ± 0.09	2.36 ± 0.13	2.39 ± 0.15	0.634	0.571	0.946
Diabetes^b^	2.31 (0.96–5.60)	1 (ref.)	0.86 (0.44–1.68)	0.75 (0.36–1.59)	–	0.029	0.257
Diabetes^c^	2.23 (0.92–5.41)	1 (ref.)	0.88 (0.45–1.72)	0.72 (0.34–1.52)	–	0.030	0.337
Diabetes^d^	2.19 (0.90–5.33)	1 (ref.)	0.87 (0.44–1.71)	0.71 (0.33–1.53)	–	0.032	0.352
High HOMA^b^	0.84 (0.44–1.60)	1 (ref.)	0.77 (0.52–1.13)	0.67 (0.44–1.03)	–	0.407	0.451
Metabolic syndrome^a^	0.96 (0.52–1.76)	1 (ref.)	1.05 (0.73–1.50)	*1.49 (1.02–2.17)*	–	0.185	0.410

Results are expressed as adjusted mean ± standard error or as odds ratio and (95 % confidence interval). Normal weight was defined as a body mass index <25 kg/m^2^; abdominal obesity was defined as a waist circumference ≥88 cm for women and ≥102 cm in men. Statistical analysis conducted using analysis of variance for continuous variables and logistic regression for categorical variables. Statistically significant odds ratios are indicated in italics. Column p value corresponds to the p value of the overall association test; column linear trend corresponds to the p value for testing a linear trend. Adjusted for: ^a^ age, smoking status and physical activity; ^b^ age, smoking status, physical activity and BMI; ^c^ age, smoking status, physical activity and waist circumference; ^d^ age, smoking status, physical activity and fat mass. Also adjusted for ^e^ antidiabetic drug treatment

^§^Statistical analysis performed on log-transformed data

### Birth weight and markers of glucose homeostasis

Bivariate associations between markers of glucose homeostasis and birth weight group are summarized in Table 2 (for women) and Table 3 (for men). Fasting glucose, insulin and HOMA levels and prevalence of diabetes and metabolic syndrome showed significant differences between birth weight categories in women (Table 2) but not in men (Table 3).

The results of the multivariate analysis of the associations between markers of glucose homeostasis and birth weight categories are summarized in Table 4 (for women) and Table 5 (for men). In women, low birth weight was associated with a higher likelihood of developing diabetes, insulin resistance (as assessed by high HOMA levels) and metabolic syndrome (Table 4). Conversely, no significant associations were found in men, with the exception that birth weights >4.0 kg were associated with a higher likelihood of developing metabolic syndrome (Table 5). Sensitivity analysis restricting to people reporting a birth weight >1.5 kg led to similar findings, low birth weight being now also significantly associated with diabetes in men (Additional file 1: Table S2a, b).

## Discussion

To our knowledge, this is the largest population-based study to assess the associations between birth weight and adult levels of markers of glucose homeostasis, obesity, leptin and adiponectin in Europe. Our findings provide some insight regarding the differential effects of low and high birth weight on diabetes and obesity markers in later life.

Here, we report higher levels in markers of glucose homeostasis in people reporting low birth weight, but also a U-shaped association between birth weight and most obesity markers, notably among women. Those findings are in agreement with the literature [1, 16, 17], and indicate that low birth weight is associated with increased risk of diabetes and obesity in later life.

### Birth weight and obesity markers

Leptin is a hormone produced by the adipose tissue and is classically increased in type 2 diabetes [18], but is mostly associated with obesity [19]. Diet early in life might regulate leptin levels [15], which in turn will influence energy intake and risk of obesity [4]. In this study, adult leptin levels decreased consistently with increased birth weight in both genders; those findings are in agreement with some studies [4, 5] but not with other smaller sized studies, none of which being population-based (Jaquet et al. (case–control study, N = 26 cases and 25 controls, age = 24 years) [20]; Melo et al. N = 165, not population-based, only women) [21]). Similarly, the leptin to fat mass ratio significantly decreased with increasing birth weight in both gender, a finding that, to our knowledge, had not been reported previously. Overall, the decrease in leptin levels and in the leptin to fat mass ratio with birth weight suggest that the mechanisms by which low or high birth weight influence the development of obesity in later life might be different. Interestingly, it has been suggested that children born with a low weight develop high leptin levels during catch-up growth, which suggests leptin resistance [22]. Hence, our data suggest that the high leptin levels observed at birth and during childhood for low birth children could actually be maintained in later life. Another possible mechanism would be adipocyte dysfunction: catch-up growth is associated with adipocyte hypertrophy in male rats [23], hypertrophic adipocytes or adipocytes from low birth weight animals have a distinct gene expression [24] and high leptin levels are more closely associated with adipose cell hypertrophy than with adipose tissue hyperplasia [25]. Nevertheless, these findings in animals are not corroborated in cultured preadipocytes isolated from adult people born with low birth weight. Indeed, these preadipocytes show reduced leptin expression and release compared to people born with a normal weight, suggesting impaired preadipocytes maturation [26]. These surprising results might be due to the fact that adult body weight was significantly lower in people born with a low weight compared to people born with a normal weight. Moreover, fat mass assessed by DEXA was similar between groups and this study was only performed in males [26]. Finally, the high leptin to fat mass ratio observed in people born with a low weight could be a sequel of malnutrition during foetal life [27]. Overall, our data suggest that low birth weight leads to increased adult leptin levels due to the association of leptin resistance and increased leptin production as assessed by the leptin to fat mass ratio.

High birth weight has also been shown to be related with obesity [28, 29], although other studies suggest that high birth weight is actually more related to lean than to fat mass (for a review, see [30]). Still, no differences in body fat percentage and fat mass were found between low and high birth weight groups in both genders, even when the analysis was restricted to obese people (not shown). Hence, the hypothesis that a high birth weight is related to an increased lean mass in later life is not confirmed by our data. Again, the precise mechanisms for the increased obesity risk among high birth weight children remain to be assessed. Maternal obesity is the most likely explanation, as it is related to foetal macrosomia and to increased risk of obesity [31, 32], although this statement has been challenged [33]. Indeed, the socio-economic position of the mother might be the strongest determinant, as it is associated with a number of risk factors for child obesity [34]. Conversely, the reasons for lower leptin and leptin to fat mass ratio in people born with a high weight can only be speculated. Increased birth weight is associated with high leptin levels [35, 36], which can be further reduced by breastfeeding [15]. The fact that participants with a high birth weight also present with the lowest leptin levels could thus be due to differences in nutrition in early life, or in adipocyte metabolism [24–26] as assessed by a lower leptin production by body fat unit. Still, further studies are needed to explain those findings.

Adiponectin is another protein secreted by adipocytes. Reduced levels of adiponectin are observed in obese people [37] and associated with an increased risk of type 2 diabetes [8]. Blood cord adiponectin levels are positively related with birth weight and BMI in newborns [7], although this statement has been challenged [9]. Indeed, a study found lower adiponectin levels in young adults born small for gestational age (SGA) [38], while another reported higher adiponectin levels in prepubertal children born SGA compared to children born with low weight adequate for gestational age [39]. In our study, no association was found between birth weight and plasma adiponectin levels, suggesting that adiponectin does not mediate the associations between low or high birth weight and diabetes or obesity risk in middle-aged people, a finding also reported by others [6, 39–41]. Further studies are needed to better understand the potential link between adiponectin and birth weight, by notably evaluating the role of catch-up growth in this process.

### Birth weight and markers of glucose homeostasis

The association of markers of glucose homeostasis with low birth weight remained after adjustment for obesity markers, suggesting that low birth weight increases diabetes risk through mechanisms that are independent of obesity or body composition, warranting further research. It has been suggested that people born with a low weight tend to have a more visceral distribution of obesity and significantly reduced muscle mass, with an increased body fat content [42]. A study suggested that low fat deposition leading to thinness at birth and during infancy results in fat acquisition during childhood, with a subsequent increase in the risk of developing type 2 diabetes [43]. Indeed, a higher waist circumference and percentage of body fat were observed in women born with a low weight, but not in men. Moreover, women born with a low weight also presented higher BMI and obesity levels. Hence, although our findings partly confirm that people born with a low weight tend to present a higher body fat content and diabetes risk, further studies are needed to better assess this point, namely regarding the differences between genders. An important confounder could be catch-up growth during the first years of life, as it is more closely linked to adult adiposity than birth weight [44–47].

### Possible impact on cardiovascular disease

Low birth weight has been shown to be significantly associated with increased all-cause mortality in both genders and with increased cardiovascular mortality in men [48]. The higher leptin levels observed among participants born with a low weight might partly contribute to this observation, as higher leptin levels have been shown to be an independent risk factor for cardiovascular disease among diabetic patients [49, 50]. Low birth weight might also impact vascular endothelium properties [51] and increases the risk of obesity and type 2 diabetes [52], probably via a common genetic mechanism [53, 54]. Further, earlier age at menarche among obese adolescents might also be an independent risk factor for the development of type 2 diabetes and cardiometabolic disease [55]. Overall, our results suggest that people born with a low weight should be counselled regarding the risks of developing diabetes and/or cardiovascular disease.

### Study limitations

Our study has several limitations. First, the participation rate was low (41 %), which might limit the generalization of the findings; yet, this participation rate is in line with other epidemiological studies [56]. In addition, the distribution of age groups 35–54 and 55–75 in the CoLaus study was comparable to the source population and there was no gender or zip code distribution difference between the source population and the CoLaus participants (not shown). Second, self-reported birth weight was only available for a subset of participants that substantially differed from participants who did not provide this information. Third, no objective validation of the reported birth weights was performed, thus leading to a possible reporting bias. Still, it has been shown that the validity of self-reported birth weight is high [57]. Fourth, no information regarding gestational age at birth was collected. Hence, it was not possible to distinguish between a low birth weight adequate for gestational age (AGA) from a low birth weight small for gestational age (SGA). This might have confounded our findings, as it has been shown that children born SGA already present with insulin resistance and higher adiponectin levels at prepubertal age, although no difference in leptin levels have been reported [39]. Finally, we could not evaluate the impact of catch-up growth in early life, as we could not record body weight gain during early infancy due to the nature of the CoLaus study.

### Conclusion

Our results suggest that people born with a low weight present a higher prevalence of diabetes and that both low and high birth weight are associated with obesity markers, compared to people born with a normal weight. The higher leptin levels and higher leptin to fat mass ratio among people born with a low weight might be related to nutritional factors during childhood, leptin resistance, or to a higher leptin production by body fat unit.

## Additional file

10.1186/s12933-016-0389-2 Additional tables.

## Abbreviations

ANOVA: analysis of variance
ATP-III: Third Adult Treatment Panel
BF: body fat
BMI: body mass index
CoLaus: cohorte Lausannoise (Lausanne cohort study)
CV: coefficient of variation
ELISA: enzyme-linked immunosorbent assay
HOMA: homeostasis model assessment index of insulin resistance
