# Supplementary material for: Low birth weight leads to obesity, diabetes and increased leptin levels in adults: the CoLaus study
Source: Cardiovasc Diabetol. 2016 May 3;15:73. doi: 10.1186/s12933-016-0389-2 (PMC4855501; doi:10.1186/s12933-016-0389-2)
Supplement: Supplementary file 1 — 10.1186/s12933-016-0389-2 Additional tables. [file 12933_2016_389_MOESM1_ESM.docx]

**Supplementary tables**

**Supplementary table 1**: characteristics of participants included and excluded from the study

|  | Included (n=2546) | Excluded (n=4187) | P-value |
| --- | --- | --- | --- |
| Women (%) | 1458 (57.3) | 2086 (49.8) | <0.001 |
| Age (years) | 50.0 ± 10.0 | 54.2 ± 10.9 | <0.001 |
| BMI categories (%) |  |  | <0.001 |
| Normal | 1336 (52.5) | 1901 (45.4) |  |
| Overweight | 866 (34.0) | 1596 (38.2) |  |
| Obese | 344 (13.5) | 687 (16.4) |  |
| Abdominal obesity (%) | 678 (26.6) | 1297 (31.0) | <0.001 |
| Physically active (%) | 1466 (57.6) | 2041 (48.8) | <0.001 |
| Smoking status (%) |  |  | 0.006 |
| Never | 1079 (42.4) | 1653 (39.5) |  |
| Former | 768 (30.2) | 1415 (33.8) |  |
| Current | 699 (27.5) | 1113 (26.6) |  |

Results are expressed as number of participants (percentage) or as average ± standard deviation. Between-group comparisons performed using chi-square for categorical variables and Student’s t-test for continuous variables. §, analysis performed on log-transformed data.

**Supplementary table 2a**: distribution of anthropometric and biological variables according to birth weight, women, multivariate adjusted. Participants with reported birth weight <1.5 kg excluded.

|  |  | **Birth weight categories (kg)** | |  |  |  |  |
| --- | --- | --- | --- | --- | --- | --- | --- |
|  | **1.5-2.5] (n=155)** | **]2.5 – 3.5] (n=891)** | **]3.5 – 4.0] (n=264)** | **]4.0+ (n=129)** | **p-value** | **Linear** | **Quadratic** |
| Anthropometry ^1^ |  |  |  |  |  |  |  |
| Height (cm) | 161 ± 1 | 164 ± 1 | 165 ± 1 | 166 ± 1 | <0.001 | <0.001 | 0.114 |
| Weight (kg) | 64.6 ± 1.0 | 64.7 ± 0.4 | 69.2 ± 0.8 | 69.8 ± 1.1 | <0.001 | <0.001 | 0.769 |
| BMI (kg/m^2^) | 24.8 ± 0.4 | 24.1 ± 0.2 | 25.4 ± 0.3 | 25.4 ± 0.4 | <0.001 | 0.094 | 0.300 |
| Waist circumference (cm) | 83.2 ± 1.0 | 81.0.0 ± 0.4 | 83.5 ± 0.7 | 83.9 ± 1.1 | 0.002 | 0.283 | 0.125 |
| Fat (% of body weight) | 33.7 ± 0.6 | 32.5 ± 0.2 | 33.6 ± 0.5 | 33.2 ± 0.6 | 0.072 | 0.899 | 0.445 |
| Fat mass (kg) | 22.5 ± 0.7 | 21.6 ± 0.3 | 24.1 ± 0.6 | 23.8 ± 0.8 | <0.001 | 0.042 | 0.671 |
| Normal weight | **0.70 (0.49 - 0.99)** | 1 (ref.) | **0.66 (0.50 - 0.88)** | **0.59 (0.40 - 0.86)** | - | 0.216 | 0.093 |
| Abdominal obesity | 1.32 (0.90 - 1.92) | 1 (ref.) | **1.40 (1.03 - 1.90)** | **1.67 (1.12 - 2.49)** | - | 0.181 | 0.136 |
| Adipokines |  |  |  |  |  |  |  |
| Leptin (ng/dL) ^1^ | 18.2 ± 1.0 | 15.5 ± 0.4 | 16.9 ± 0.7 | 16 ± 1.1 | 0.260 § | 0.244 § | 0.418 § |
| Leptin (ng/dL) ^2^ | 17.3 ± 0.7 | 16.3 ± 0.3 | 15.8 ± 0.5 | 13.8 ± 0.8 | 0.007 § | 0.002 § | 0.358 § |
| Leptin (ng/dL) ^3^ | 17.0 ± 0.8 | 16.1 ± 0.3 | 16.1 ± 0.6 | 14.4 ± 0.8 | 0.128 § | 0.035 § | 0.442 § |
| Leptin (ng/dL) ^4^ | 17.6 ± 0.7 | 16.2 ± 0.3 | 15.6 ± 0.5 | 14.0 ± 0.8 | 0.009 § | 0.002 § | 0.665 § |
| Leptin/fat mass ratio ^2^ | 0.79 ± 0.04 | 0.73 ± 0.02 | 0.69 ± 0.03 | 0.62 ± 0.04 | 0.019 | 0.002 | 0.839 |
| Adiponectin (µg/dL) ^1^ | 11.3 ± 0.7 | 12.3 ± 0.3 | 11.9 ± 0.5 | 12.2 ± 0.8 | 0.558 § | 0.232 § | 0.872 § |
| Adiponectin (µg/dL) ^2^ | 11.5 ± 0.7 | 12.1 ± 0.3 | 12.2 ± 0.5 | 12.5 ± 0.8 | 0.425 § | 0.104 § | 0.793 § |
| Adiponectin (µg/dL) ^3^ | 11.5 ± 0.7 | 12.1 ± 0.3 | 12.2 ± 0.5 | 12.5 ± 0.8 | 0.541 § | 0.149 § | 0.791 § |
| Adiponectin (µg/dL) ^4^ | 11.3 ± 0.7 | 12.1 ± 0.3 | 12.2 ± 0.5 | 12.5 ± 0.8 | 0.407 § | 0.090 § | 0.985 § |
| Diabetes markers |  |  |  |  |  |  |  |
| Glucose (mmol/L) ^2,a^ | 5.37 ± 0.06 | 5.28 ± 0.02 | 5.26 ± 0.04 | 5.18 ± 0.06 | 0.136 | 0.019 | 0.926 |
| Insulin (μU/mL) ^2,a^ | 8.2 ± 0.4 | 7.5 ± 0.2 | 8.0 ± 0.3 | 6.5 ± 0.4 | 0.012 | 0.008 | 0.211 |
| HOMA ^2,a^ | 2.00 ± 0.1 | 1.83 ± 0.04 | 1.95 ± 0.08 | 1.50 ± 0.12 | 0.006 | 0.004 | 0.120 |
| Diabetes ^2^ | **2.94 (1.29 - 6.69)** | 1 (ref.) | 1.42 (0.58 - 3.50) | 1.91 (0.72 - 5.09) | - | 0.581 | 0.057 |
| Diabetes ^3^ | 2.36 (0.93 - 6.00) | 1 (ref.) | 0.87 (0.31 - 2.42) | 1.86 (0.65 - 5.32) | - | 0.642 | 0.043 |
| Diabetes ^4^ | **2.70 (1.09 - 6.67)** | 1 (ref.) | 0.69 (0.24 - 1.98) | 1.17 (0.38 - 3.57) | - | 0.139 | 0.066 |
| High HOMA ^2^ | 1.57 (0.99 - 2.47) | 1 (ref.) | 1.15 (0.76 - 1.74) | 0.86 (0.48 - 1.56) | - | 0.123 | 0.693 |
| Metabolic syndrome ^1^ | **1.76 (1.13 - 2.74)** | 1 (ref.) | 1.13 (0.75 - 1.70) | 1.05 (0.62 - 1.79) | . | 0.147 | 0.201 |

Results are expressed as adjusted mean ± standard error or as odds ratio and (95% confidence interval). Normal weight was defined as a body mass index <25 kg/m^2^; abdominal obesity was defined as a waist circumference ≥88 cm for women and ≥102 cm in men. Statistical analysis conducted using analysis of variance for continuous variables and logistic regression for categorical variables. Statistically significant odds ratios are indicated in bold. Column p-value corresponds to the p-value of the overall association test; column linear trend corresponds to the p-value for testing a linear trend. Adjusted for: ^1^age, smoking status and physical activity; ^2^age, smoking status, physical activity and BMI; ^3^age, smoking status, physical activity and waist circumference; ^4^age, smoking status, physical activity and fat mass. Also adjusted for ^a^ antidiabetic drug treatment. §, statistical analysis performed on log-transformed data.

**Supplementary table 2b**: distribution of anthropometric and biological variables according to birth weight, men, multivariate adjusted. Participants with reported birth weight <1.5 kg excluded.

|  |  | **Birth weight categories (kg)** | |  |  |  |  |
| --- | --- | --- | --- | --- | --- | --- | --- |
|  | **1.5-2.5] (n=68)** | **]2.5 – 3.5] (n=556)** | **]3.5 – 4.0] (n=266)** | **]4.0+ (n=193)** | **p-value** | **Linear** | **Quadratic** |
| Anthropometry ^1^ |  |  |  |  |  |  |  |
| Height (cm) | 175 ± 1 | 176 ± 1 | 178 ± 1 | 179 ± 1 | <0.001 | <0.001 | 0.291 |
| Weight (kg) | 80.7 ± 1.6 | 80.0 ± 0.5 | 84.6 ± 0.8 | 86.9 ± 0.9 | <0.001 | <0.001 | 0.152 |
| BMI (kg/m^2^) | 26.2 ± 1.5 | 26.0 ± 0.2 | 26.8 ± 0.2 | 27.1 ± 0.3 | <0.001 | 0.027 | 0.347 |
| Waist circumference (cm) | 94.7 ± 1.3 | 93.7 ± 0.4 | 96.1 ± 0.6 | 97.3 ± 0.8 | <0.001 | 0.023 | 0.189 |
| Fat (% of body weight) | 23.5 ± 0.6 | 22.4 ± 0.2 | 23.3 ± 0.3 | 23.4 ± 0.4 | 0.025 | 0.770 | 0.121 |
| Fat mass (kg) | 19.3 ± 0.9 | 18.3 ± 0.3 | 20.2 ± 0.4 | 20.9 ± 0.5 | <0.001 | 0.031 | 0.149 |
| Normal weight | 0.81 (0.48 - 1.38) | 1 (ref.) | **0.71 (0.52 - 0.96)** | **0.47 (0.33 - 0.68)** |  | 0.033 | 0.075 |
| Abdominal obesity | 0.97 (0.51 - 1.84) | 1 (ref.) | 1.27 (0.89 - 1.82) | **1.72 (1.17 - 2.52)** |  | 0.065 | 0.494 |
| Adipokines |  |  |  |  |  |  |  |
| Leptin (ng/dL) ^1^ | 10.2 ± 1.0 | 8.6 ± 0.3 | 8.2 ± 0.5 | 8.7 ± 0.6 | 0.635 § | 0.517 § | 0.201 § |
| Leptin (ng/dL) ^2^ | 10.3 ± 0.8 | 9.0 ± 0.3 | 8.0 ± 0.4 | 7.9 ± 0.5 | 0.090 § | 0.042 § | 0.529 § |
| Leptin (ng/dL) ^3^ | 10.2 ± 0.8 | 9.0 ± 0.3 | 8.0 ± 0.4 | 7.8 ± 0.5 | 0.055 § | 0.032 § | 0.693 § |
| Leptin (ng/dL) ^4^ | 10.0 ± 0.8 | 9.1 ± 0.3 | 7.8 ± 0.4 | 7.7 ± 0.5 | 0.016 § | 0.021 § | 0.609 § |
| Leptin/fat mass ratio ^2^ | 0.46 ± 0.05 | 0.45 ± 0.02 | 0.39 ± 0.02 | 0.38 ± 0.03 | 0.039 | 0.069 | 0.981 |
| Adiponectin (µg/dL) ^1^ | 8.4 ± 0.7 | 7.2 ± 0.2 | 7.1 ± 0.4 | 7.3 ± 0.4 | 0.845 § | 0.693 § | 0.580 § |
| Adiponectin (µg/dL) ^2^ | 8.3 ± 0.7 | 7.1 ± 0.2 | 7.1 ± 0.4 | 7.4 ± 0.4 | 0.890 § | 0.978 § | 0.505 § |
| Adiponectin (µg/dL) ^3^ | 8.3 ± 0.7 | 7.1 ± 0.2 | 7.1 ± 0.4 | 7.5 ± 0.4 | 0.862 § | 0.989 § | 0.466 § |
| Adiponectin (µg/dL) ^4^ | 8.4 ± 0.7 | 7.1 ± 0.2 | 7.2 ± 0.4 | 7.4 ± 0.4 | 0.875 § | 0.973 § | 0.458 § |
| Diabetes markers |  |  |  |  |  |  |  |
| Glucose (mmol/L) ^2,a^ | 5.65 ± 0.12 | 5.65 ± 0.04 | 5.68 ± 0.06 | 5.62 ± 0.07 | 0.928 | 0.898 | 0.708 |
| Insulin (μU/mL) ^2,a^ | 9.6 ± 0.8 | 9.8 ± 0.3 | 8.8 ± 0.4 | 9.1 ± 0.5 | 0.195 | 0.348 | 0.981 |
| HOMA ^2,a^ | 2.55 ± 0.26 | 2.55 ± 0.09 | 2.36 ± 0.13 | 2.39 ± 0.15 | 0.620 | 0.479 | 0.943 |
| Diabetes ^2^ | **2.57 (1.11 - 5.93)** | 1 (ref.) | 1.15 (0.61 - 2.14) | 0.96 (0.47 - 1.98) |  | 0.067 | 0.194 |
| Diabetes ^3^ | 2.36 (0.96 - 5.78) | 1 (ref.) | 0.88 (0.45 - 1.72) | 0.72 (0.34 - 1.53) |  | 0.024 | 0.295 |
| Diabetes ^4^ | 2.33 (0.95 - 5.73) | 1 (ref.) | 0.87 (0.45 - 1.71) | 0.71 (0.33 - 1.53) |  | 0.025 | 0.304 |
| High HOMA ^2^ | 1.07 (0.58 - 1.95) | 1 (ref.) | 0.91 (0.64 - 1.31) | 0.89 (0.60 - 1.33) |  | 0.540 | 0.916 |
| Metabolic syndrome ^1^ | 0.96 (0.51 - 1.78) | 1 (ref.) | 1.05 (0.73 - 1.50) | **1.49 (1.02 - 2.17)** |  | 0.189 | 0.429 |

Results are expressed as adjusted mean ± standard error or as odds ratio and (95% confidence interval). Normal weight was defined as a body mass index <25 kg/m^2^; abdominal obesity was defined as a waist circumference ≥88 cm for women and ≥102 cm in men. Statistical analysis conducted using analysis of variance for continuous variables and logistic regression for categorical variables. Statistically significant odds ratios are indicated in bold. Column p-value corresponds to the p-value of the overall association test; column linear trend corresponds to the p-value for testing a linear trend. Adjusted for: ^1^age, smoking status and physical activity; ^2^age, smoking status, physical activity and BMI; ^3^age, smoking status, physical activity and waist circumference; ^4^age, smoking status, physical activity and fat mass. Also adjusted for ^a^ antidiabetic drug treatment. §, statistical analysis performed on log-transformed data.
